# Supplementary figures and images for: Upper-critical solution temperature (UCST) polymer functionalized nanomedicine for controlled drug release and hypoxia alleviation in hepatocellular carcinoma therapy
Source: PLoS One. 2023 Aug 25;18(8):e0290237. doi: 10.1371/journal.pone.0290237 (PMC10456220; doi:10.1371/journal.pone.0290237)

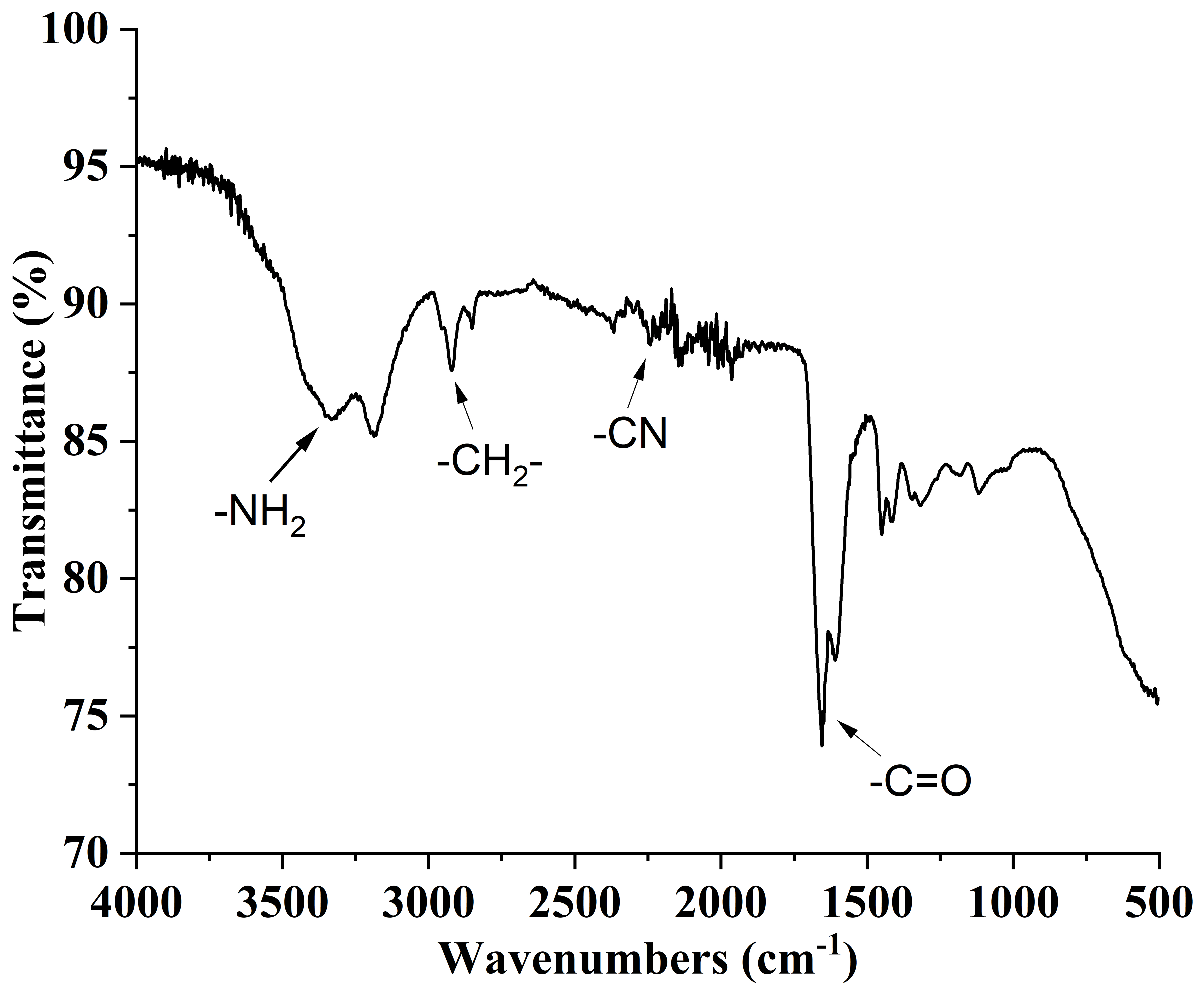

Supplement: S1 Fig — (PNG) [file pone.0290237.s001.png]

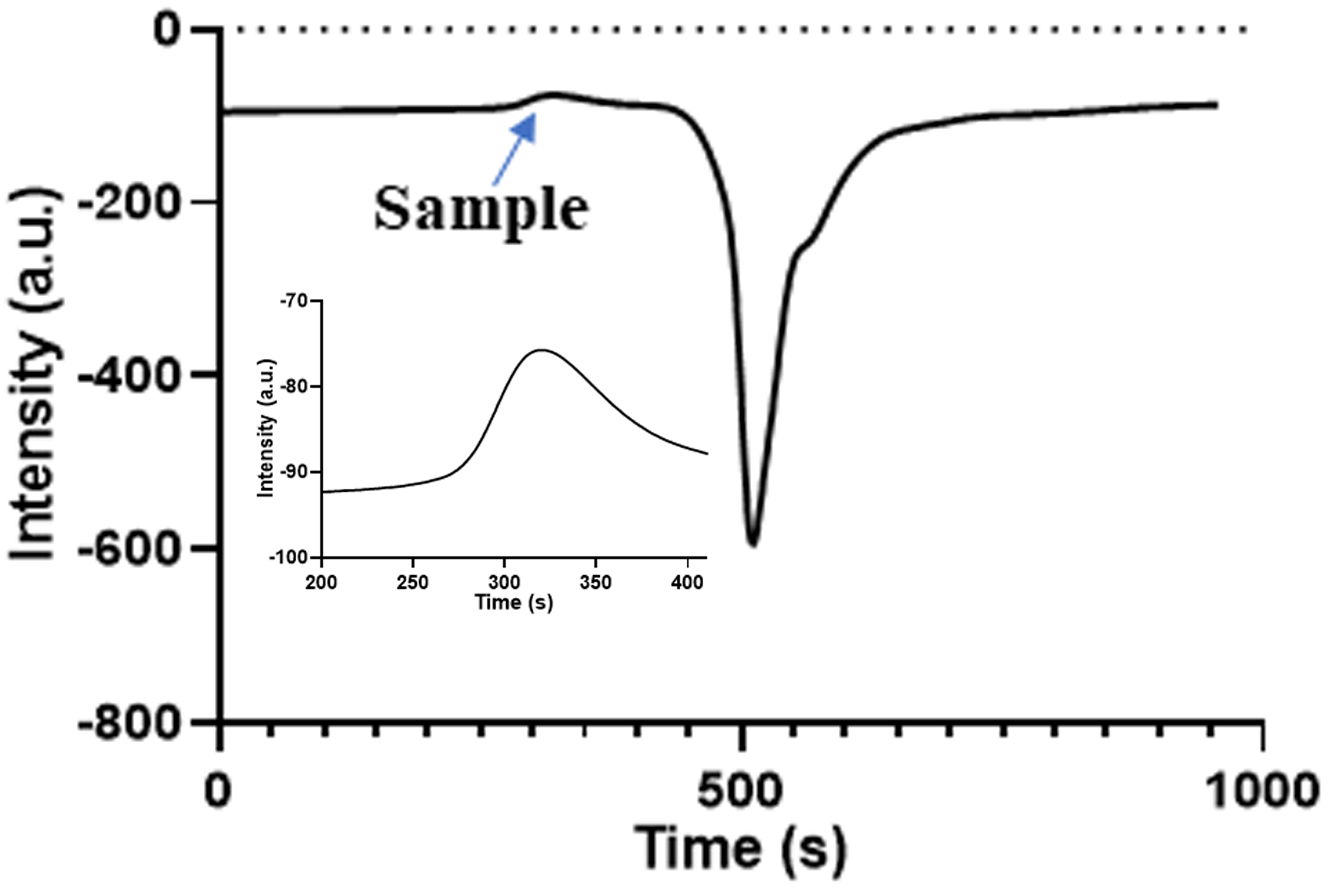

Supplement: S2 Fig — (PNG) [file pone.0290237.s002.png]

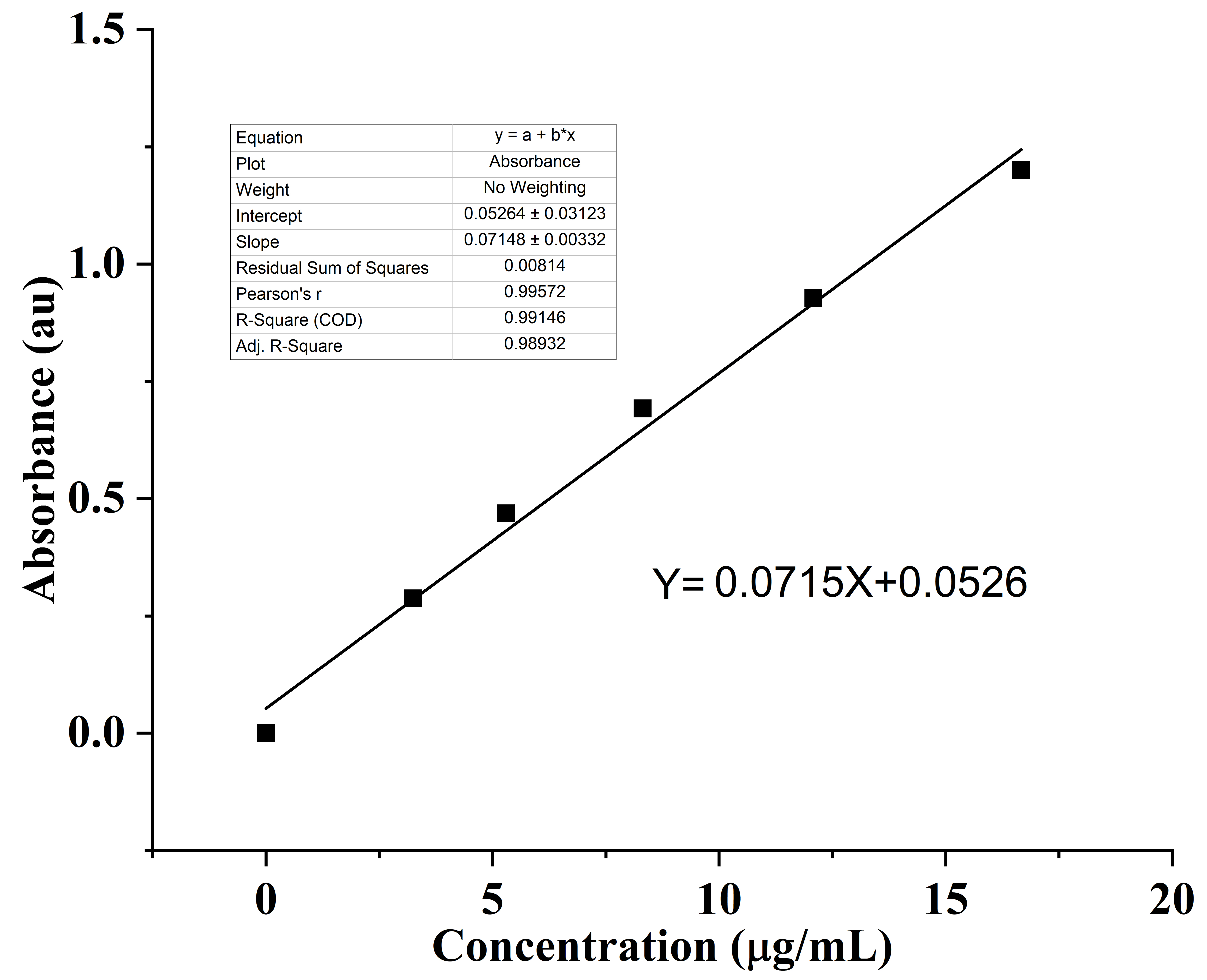

Supplement: S3 Fig — (PNG) [file pone.0290237.s003.png]

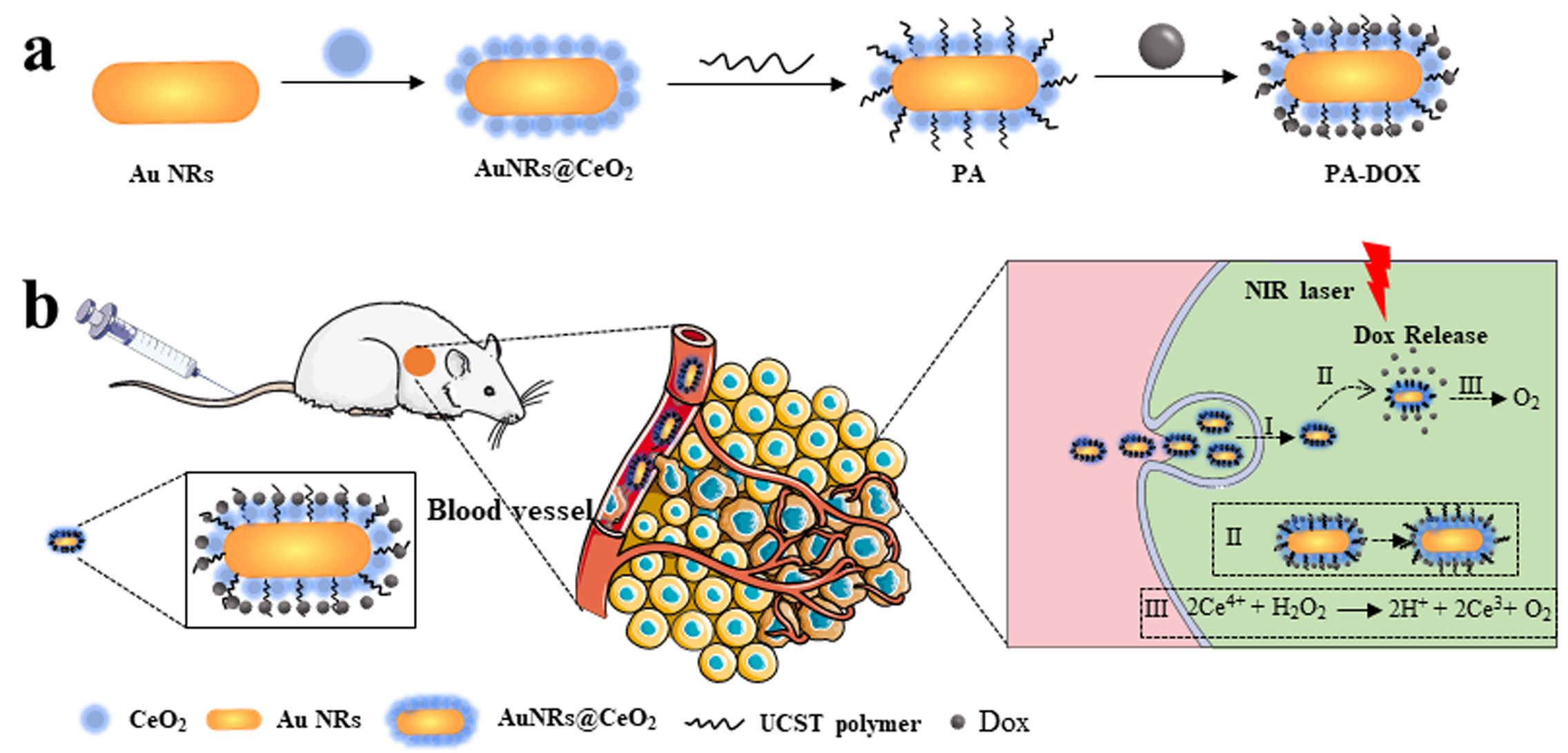

Supplement: S1 Striking image — (TIF) [file pone.0290237.s004.tif]
