# Supplementary material for: Upper-critical solution temperature (UCST) polymer functionalized nanomedicine for controlled drug release and hypoxia alleviation in hepatocellular carcinoma therapy
Source: PLoS One. 2023 Aug 25;18(8):e0290237. doi: 10.1371/journal.pone.0290237 (PMC10456220; doi:10.1371/journal.pone.0290237)
Supplement: Attachment — Submitted filename: Reviewers comments.docx [file pone.0290237.s005.docx]

This paper presents a type of drug-loaded nanoparticles PA-DOX for the treatment of liver cancer, in which hydrophobic gold nanoparticles are modified with Upper-critical solution temperature (UCST) polymer P(AAm-co-AN), CeO_2_ and DOX were loaded in the nanosystem. When the system is irradiated by NIR light, the local heating was produced and triggered the phase change of the P(AAm-co-AN) polymer, simultaneously, CeO_2_ and DOX were released. Released CeO_2_ can react with internal H_2_O_2_ to generate O_2_ for relieving the hypoxia of tumor and improving the anticancer effect of DOX. This system seems interesting, however, there are many problems in this manuscript, and some results lack of deep discussion. In my opinion, this manuscript needs major revisions before it can be reconsidered for publication in PLOS ONE, and the following concerns should be well addressed in the revision:

1．Please confirm whether hydrogen peroxide and H_2_O_2_ in the sentence “CeO_2_ can decompose hydrogen peroxide to H_2_O_2_ and O_2_” of abstract are the same substance. (Aren't hydrogen peroxide and H_2_O_2_ the same substance?)

2．A reference should be cited for the preparation of AuNRs.

3. To check the mean of the sentence“The polymer layer of P(AAm-co-AN) appeared to collapse at low temperatures to confine the release of DOX” in manuscript (page 3, lines 29-30).

4. The written form of PA-DOX should be unified in the manuscript, P(AAm-co-AN)-AuNRs@CeO_2_-DOX or P(AAm-co-AN)/AuNRs@CeO_2_-DOX?

5．In formula (2), 2 in H_2_O should be subscripted; the 4 section, 2 in CeO_2_ should be subscripted.

6．The drug loading content was calculated to be 7.1%, why is the drug loading content in such a low level?

7．The photothermal performance experiments of PA-DOX should be provided in experiment section.

8．The drug DOX in the nanodrug only releases when the lesion site is irradiated, and the release stops when the temperature in the lesion site drops to the physiological temperature. That means the treatment process is troublesome, otherwise, the drugs loaded cannot be fully utilized. How to explain that?

9. To check the sentence “To investigate the oxygen generation ability of PA-DOX, AuNRs, Au@CeO_2_ or PA-DOX, respectively, all these samples were dispersed in the H_2_O_2_ solution with or without the laser irradiation.” in 3.4 section.

10. All pictures in the manuscript are blurred. Please provide pictures with high resolution.
